# Long-term outcomes of intraoperative triamcinolone injection versus postoperative oral prednisolone in congenital cataract surgery

**DOI:** 10.5935/0004-2749.2021-0514

**Published:** 2023-03-08

**Authors:** Bruna Vieira Ventura, Mariana Zaira Ribeiro, Nayara Rayanne Bezerra Caldas, Larissa Ventura, Polyana Marinho, Rodrigo Pessoa Cavalcanti Lira, Camilla Silva da Rocha, Marcelo Carvalho Ventura

**Affiliations:** 1 Fundação Altino Ventura, Recife, PE, Brazil; 2 Hospital de Olhos de Pernambuco, Recife, PE, Brazil; 3 Universidade Federal de Pernambuco, Recife, PE, Brazil

**Keywords:** Congenital cataract, Triamcinolone, Prednisolone, Steroids, Postoperative complications, Children, Cataract, Catarata congênita, Triancinolona, Prednisolona, Esteroides, Complicações pós-operatórias, Criança

## Abstract

**Purpose:**

To compare the long-term ocular findings of children that were operated of congenital cataract before the age of two and that received an intraoperative intracameral triamcinolone injection or used postoperative oral prednisolone to modulate ocular inflammation.

**Methods:**

All patients who had previously participated in a clinical trial that analyzed the 1-year surgical outcomes of congenital cataract surgery utilizing intracameral triamcinolone (study group) or oral prednisolone (control group) were eligible to participate in this prospective cohort research. Patients’ medical records were reviewed, and the children underwent a complete ophthalmologic exam on final follow-up. Biomicroscopic findings, intraocular pressure, central corneal thickness, the need for additional surgical interventions, and findings compatible with glaucoma were the primary end measures.

**Results:**

Twenty-six eyes (26 patients) were included (study group = 11 eyes; control group = 15 eyes). The mean follow--up was 8.2 ± 1.2 years and 8.1 ± 1.7 years in the study and control groups, respectively (p=0.82). All eyes presented a centered intraocular lens. There was no statistically significant difference between the groups with regards to the presence of posterior synechia (p=0.56), intraocular pressure (p=0.49), or central corneal thickness (p=0.21). None of the eyes fulfilled the glaucoma diagnostic criteria, presented secondary visual axis obscuration, or were reoperated.

**Conclusion:**

The long--term ocular findings of children that underwent congenital cataract surgery and received an intraoperative intracameral triamcinolone injection were similar to those that used postoperative oral prednisolone to modulate ocular inflammation. This suggests that intracameral triamcinolone may substitute oral prednisolone in congenital cataract surgery, facilitating the postoperative treatment regimen and compliance.

## Abstract (pt)

**Objetivo:**

Comparar os achados oculares em longo prazo de crianças que se submeteram à cirurgia de catarata congênita antes dos dois anos de idade e receberam uma injeção intracameral de triancinolona no intraoperatório ou usaram prednisolona oral no pós-operatório para modular a inflamação ocular.

**Métodos:**

Neste estudo prospectivo de coorte, todos os pacientes que participaram de um ensaio clínico anterior, que analisou os resultados cirúrgicos de 1 ano da cirurgia de catarata congênita usando triancinolona intracameral (Grupo de Estudo) ou prednisolona oral (Grupo Controle), eram elegíveis para participar. Os prontuários médicos dos pacientes foram revisados e as crianças foram submetidas a um exame oftalmológico completo no acompanhamento final. As principais medidas de desfecho foram: achados biomicroscópicos, pressão intraocular, espessura central da córnea, a necessidade de intervenções cirúrgicas adicionais e achados compatíveis com glaucoma.

**Resultados:**

Vinte e seis olhos (26 pacientes) foram incluídos (Grupo de Estudo = 11 olhos; Grupo de Controle = 15 olhos). O seguimento médio foi de 8,2 ± 1,2 anos e 8,1 ± 1,7 anos nos Grupos de Estudo e Controle, respectivamente (p=0,82). Todos os olhos apresentavam lente intraocular centrada. Não houve diferença estatisticamente significativa entre os grupos com relação à presença de sinéquia posterior (p=0,56), pressão intraocular (p=0,49) ou espessura central da córnea (p=0,21). Nenhum dos olhos preencheu os critérios diagnósticos para glaucoma, apresentou opacificação secundária do eixo visual ou foi reoperado.

**Conclusão:**

Os achados oculares em longo prazo de crianças que se submeteram à cirurgia de catarata congênita e receberam uma injeção intracameral de triancinolona no intraoperatório foram semelhantes aos que usaram prednisolona oral no pós-operatório para modular a inflamação ocular, sugerindo que a triancinolona intracameral pode substituir a prednisolona oral na cirurgia de catarata congênita, facilitando o tratamento pós-operatório e a adesão ao mesmo.

## INTRODUCTION

Over the years, topical and systemic steroids have been used to suppress postoperative inflammation in children undergoing congenital cataract surgery^(1-3)^. However, two concerns prompted the quest for other safe and effective steroid delivery methods: 1) the need for frequent doses to compensate for rapid drug absorption - and the possible side effects associated with this^(4,5)^; 2) the need to facilitate treatment regimen to ensure compliance and to decrease the risk of postoperative complications, such as fibrinous uveitis, formation of pupillary membrane, secondary visual axis opacification, and intraocular lens (IOL) dislocation^(6,7)^.

Triamcinolone acetonide is a prolonged steroid that was first used in children to aid in the visualization of the vitreous body and to ensure a thorough, complete anterior vitrectomy^(8,9)^. We originally published our findings on intracameral triamcinolone injection at the end of congenital cataract surgery to modulate postoperative inflammation in 2012^(10)^. In the first year after surgery, neither intraocular pressure (IOP) nor central corneal thickness (CCT) increased significantly. Subsequently, in a randomized controlled trial, we found that intracameral triamcinolone injection was as effective as oral steroids in modulating ocular inflammation in a short follow-up period, with the advantage of relieving caregivers’ burden with the postoperative regimen^(2)^. Since it is important to assess the results of new treatment approaches over time, we aimed to compare the long--term ocular findings of children that underwent congenital cataract surgery younger than two years of age and received an intraoperative intracameral triamcinolone injection or used postoperative oral prednisolone.

## METHODS

This prospective cohort study was conducted at the *Fundação Altino Ventura*, in Recife, Brazil. The study protocol followed the guidelines of the Declaration of Helsinki and was approved by the Ethics Committee of the *Fundação Altino Ventura*. Written informed consent was obtained from the patients’ guardian before their inclusion in the study.

All 60 children who participated in our previous randomized clinical trial^(2)^ were deemed eligible to participate in the present one. In this past study, we compared the surgical outcomes of patients aged <2 years who received an intracameral triamcinolone injection at the end of cataract surgery (study group=31 eyes of 31 children) or who used postoperative oral prednisolone (control group=29 eyes of 29 children) to modulate intraocular inflammation.

Briefly, the surgery in both groups consisted of phacoaspiration, endocapsular tension ring insertion, posterior capsulotomy, anterior vitrectomy, and IOL implantation^(2)^. A foldable hydrophobic acrylic Type 7B IOL (Alcon, Inc.) was implanted in the capsular bag of all patients.

The doses of preservative-free triamcinolone (Triancinolona Ophthalmos, Laboratório Ophthalmos, São Paulo, SP, Brazil) used was 1.2 mg/0.03 mL intraoperatively and of prednisolone was 1 mg/kg/day orally for the first 15 days after surgery, half of this dose was given on the third week, and one-fourth on the fourth week. All eyes also received a subconjunctival injection of 0.3 mL of 4% dexamethasone, and 0.5% moxifloxacin drops, four times daily, for 10 days; 1% tropicamide diluted 1:1 with artificial tears, twice daily, for 10 days; 0.5% betaxolol twice daily for 30 days, and 1% prednisolone acetate every 3 h daily for 1 week, which was gradually tapered over the next 6 weeks.

Patients were recruited for the current study via telephone or telegram. When these options failed to receive any response, we contacted local social workers via phone to help locate the patient and their caregivers. The children were then invited to perform a complete ophthalmological examination at the *Fundação Altino Ventura*. In addition, the patients’ records were reviewed to obtain preoperative and postoperative data, including the need for further surgical interventions along the years of follow-up.

A complete ophthalmological examination was performed in all patients included in the study. In the slit lamp examination, the following findings were specifically assessed: the presence of synequia; cell deposits on the IOL; visually significant secondary visual axis opacification (VAO) (defined as the reduction in the red reflex during retinoscopy), and IOL centration.

A Perkins applanation tonometer was used to measure the IOP under sedation, immediately before starting the surgery and then 1 year after the procedure^(2)^. In the current study, as the children were older, a Goldmann applanation tonometer was used during consultation. Glaucoma was defined as an IOP of >21 mmHg on the final follow-up in addition to at least one of the following criteria: a) an increase of >0.2 in the cup-to-disc ratio when compared to that in the preoperative assessment; b) the presence of corneal abnormalities on the slit lamp examination (i.e., Haab striae, corneal edema, increased corneal diameter). Moreover, the use of continuous medication or having undergone glaucoma surgery for IOP control at any time point after the surgery.

Furthermore, the following ancillary examinations were also performed: central pachymetry (DGH 4000B, DGH Technology, Inc., Exton, PA, USA), noncontact specular microscopy (SP3000P, Topcon Corp., Tokyo, Japan), and macular optical coherence tomography (Optovue Inc., Fremont, USA).

The main outcome measures of this study were the slit lamp findings, IOP, CCT, central corneal endothelial cell density, central foveal thickness, the need for additional surgical interventions, and the fulfillment of the criteria for glaucoma.

We excluded children who could not be reached despite all our efforts and those who were not present for the complete ophthalmological examination. Further-more, we excluded those who did not comply with the routine ocular examinations after a year of adequate follow-up, as requested by the ophthalmologist.

### Statistical analysis

Statistical analysis was performed using SPSS version 24.0 (SPSS, Inc., Chicago, IL, USA). Continuous variables were expressed as the mean ± standard deviation as well as the maximal and minimal values. Categorical variables were expressed as the absolute and relative frequencies. Categorical data were compared using Chi-square and Fisher’s exact test. Student’s *t*-test for paired data was used to compare the long-term follow-up results to those on the preoperatory and a year after the procedure. Student’s *t*-test for unpaired data were used for between-group comparisons. P<0.05 was considered to indicate statistical significance.

## RESULTS

**Figure 1:**
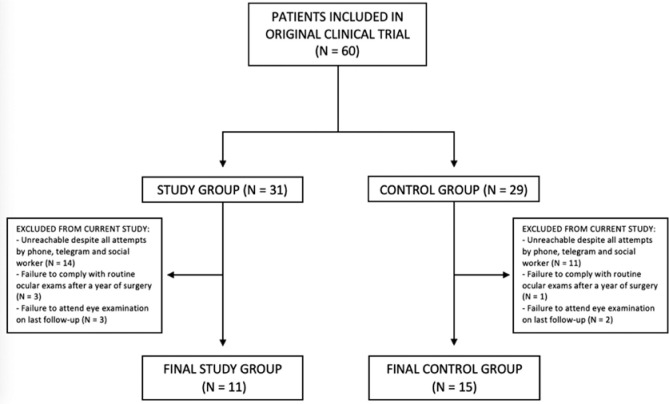
Consort flow diagram illustrating eligible and enrolled patients, along with the reasons for exclusions.

The study included twenty-six patients (26 eyes): 11 from the study group and 15 from the control group (Figure 1). In the study and control groups, patients’ mean age at surgery was 10.9 ± 5.4 months (range, 4-23 months) and 10.9 ± 6.1 months (range, 2-22 months), respectively (p0.99). In the study group, the patients’ mean age at the last follow-up visit was 8.4 ± 1.2 years (range, 6-10 years), and there were 6 (54.5%) male patients. In the control group, the patients’ mean age at the last follow-up visit was 8.3 ± 2.0 years (range, 6-11 years), and there were 9 (60.0%) male patients. Age and gender distributions were similar between both groups (p=0.82 and p=1.0, respectively). Mean follow-up was 8.2 ± 1.2 years (range, 6-10 years) in the study group and 8.1 ± 1.7 years (range, 5-11 years) in the control group (p=0.82).

At the slit lamp exam, two (18.2%) of the study group’s eyes and one (6.7%) eye of the control group presented posterior synechia (p=0.56). The synechia involved less than 3 clock hours and was evidenced only after pupillary dilation. Mild cell deposits on the IOL were found in three (27.3%) of the study group’s eyes and six (40.0%) eyes of the control group, but it did not diminish the red reflex (p=0.68). None of the eyes in either group presented secondary VAO or were reoperated during the follow-up period. Furthermore, all had a centered IOL until the last visit.

Table 1 shows the IOP and CCT data in the study and control groups before surgery, one year later, and on the final follow-up examination. There was no statistically significant difference between the groups for either IOP or CCT preoperatively (p=0.95 and p=0.27, respectively), one year after surgery (p=0.28 and p=0.56, respectively), or on the final examination (p=0.49 and p=0.21, respectively).

**Table 1:** Intraocular pressure and central corneal thickness in the study and control groups during the preoperative period, 1 year after the surgery, and on the final follow-up examination

Parameter	Study group n=11 eyes	Control group n=15 eyes	p-value^^*^^
	Mean ± SD (range)	Mean ± SD (range)	
IOP (mmHg)			
Preoperative	8.6 ± 1.6 (6-11)	8.5 ± 2.8 (4-12)	0.95
1 year postoperative	8.4 ± 2.2 (4-12)	9.7 ± 3.5 (5-18)	0.28
Final follow-up	15.0 ± 3.7 (10-20)	14.0 ± 3.1 (9-19)	0.49
CCT (µm)			
Preoperative	562.4 ± 39.9 (496-633)	544.2 ± 34.8 (467-590)	0.27
1-year postoperative	557.2 ± 16.3 (533-578)	546.3 ± 37.6 (466-589)	0.56
Final follow-up	597.0 ± 39.6 (552-692)	576.4 ± 39.4 (500-635)	0.21

SD= standard deviation; CCT= central corneal thickness; IOP= intraocular pressure.

* Student’s *t*-test.

**Figure 2:**
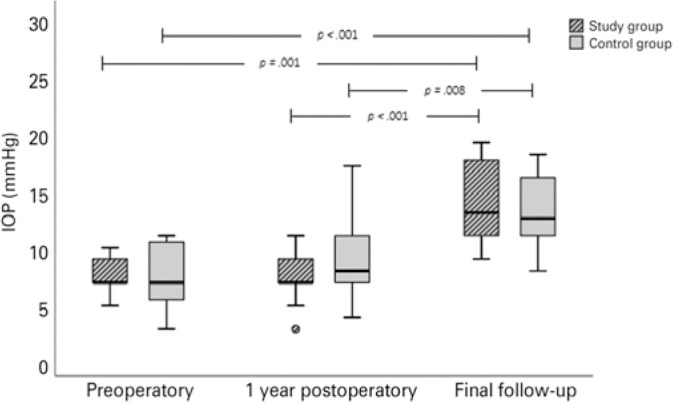
Intraocular pressure (IOP) in the study and control groups in the three studied timepoints. In both groups, IOP was statistically higher on the last follow-up visit when compared to that during the preoperatory measurement and with that 1 year after the procedure.

IOP was statistically higher in both groups on the last follow-up visit as compared to the preoperatory measurement (p=0.001 in the study group; p<0.001 in the control group) and 1 year after the procedure (p<0.001 in the study group; p=0.008 in the control group) (Figure 2). Neither group’s eyes had an IOP ≥21 mmHg on the last visit, nor did they require medication or glaucoma surgery for IOP control. When compared to the preoperative examination, the only glaucoma diagnostic criteria presented by one eye from each group (9.1% in the study group; 6.7% in the control group) was an increase of more than 0.2 in the cup-to--disc ratio (p=1.00). Thus, these two eyes did not fulfill the diagnostic criteria for glaucoma; they were designated glaucoma suspects. They had undergone a congenital cataract surgery at 23 and 8 months of age and had an IOP of 14 and 19 mmHg at the final follow-up visit (study and control groups, respectively).

**Figure 3:**
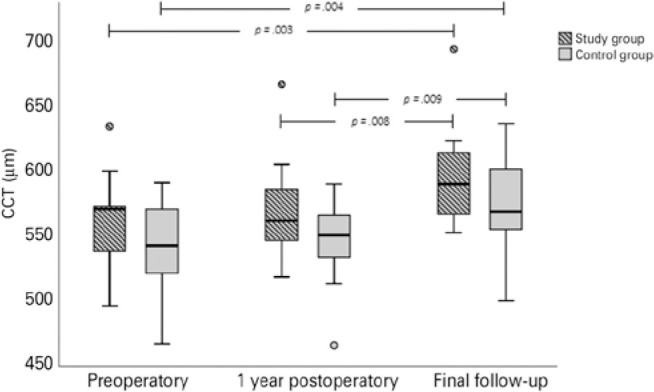
Central corneal thickness (CCT) in the study and control groups at the three studied timepoints. In both groups, CCT was statistically thicker on the last follow-up visit when compared to that at the time of preoperatory measurement and with that 1 year after the procedure.

When the CCT on the final follow-up visit was compared to that of the preoperatory, there was a significant increase in both groups (p=0.003 in the study group; p=0.004 in the control group) (Figure 3). The same was observed when analyzing the central pachymetry obtained on the final visit to that obtained 1 year after surgery (p=0.008 in the study group; p=0.009 in the control group).

The study group’s mean endothelial cell count was 2988.4 ± 496.9 cells/mm^2^ (range, 2226-3516 cells/mm^2^), whereas the control group’s was 3323.7 ± 494.3 cells/mm^2^ (range, 2575 to 4132 cells/mm^2^) (p=0.184). The mean central foveal thickness in the study and control groups was 254.2 ± 34.1 µm (range, 213 to 313 µm) and 287.5 ± 56.5 µm (range, 179 to 393 µm), respectively (p=0.119).

## DISCUSSION

Congenital cataract surgery has several challenges, including efficient suppression of the procedure’s enhanced inflammatory response^(3,7,11-13)^. Steroids play a key role in achieving this. We previously reported that a year after surgery, an intraoperative injection of 1.2 mg of intracameral triamcinolone is as effective as oral prednisolone in modulating postoperative inflammation in children younger than two years of age^(2)^. The current study assesses and compares these children’s long-term ocular findings, which is important for determining the long-term safety and efficacy of triamcinolone usage in the pediatric population.

The study and control groups exhibited identical biomicroscopic findings after a mean of 8 years of surgery, similarly to our previous paper reporting the 1-year follow-up results^(2)^. There was no statistically significant difference between the groups in terms of IOL cell deposits (all of which were mild and did not diminish the red reflex) or the prevalence of posterior synechiae. Furthermore, none of the eyes presented secondary VAO or were reoperated.

Previous studies on triamcinolone usage in pediatric cataracts have a limited follow-up^(2,8-10,13)^. Several steroid regimes were utilized in the trials that evaluated the long-term findings of cataract surgery in children. Vasavada et al.^(1)^ reported a 27.6% prevalence of posterior synechiae 5 years after surgery in eyes treated only with topical corticosteroids (6 times a day, tapered off gradually over 3 months) associated with systemic or subconjunctival steroids based on the surgeon’s clinical judgment, whereas we found an 18.2% prevalence in our study group and 6.7% in our control group. Their prevalence of secondary VAO requiring surgery was 10.3%, whereas our sample had none. Solebo et at.^(14)^ reported a 45% rate of secondary VAO requiring reintervention 5 years postoperatively. Their steroid regimen varied: the majority of eyes received only topical steroids at varying frequencies, but a minority of patients additionally received systemic corticosteroids. Interestingly, they found that intensive topical steroid treatment and the implantation of a 3-piece IOL were independent factors associated with a decreased risk of secondary VAO^(14)^. The implantation of a 3-piece IOL in all our patients, as well as our steroid regimen, may account for the disparity in reoperation rates owing to VAO observed in our paper^(1,14)^.

On the last follow-up examination, all eyes in our series had a centered IOL. We insert an endocapsular tension ring as part of our routine surgical technique to avoid capsular bag ovalization despite the implantation of a 3-piece IOL in pediatric eyes and to prevent capsular asymmetry after ocular healing, which can result from excessive capsular fibrosis and irregular shrinkage. These are the most common causes of IOL decentration in children^(15)^.

Our study and control groups did not statistically differ with regards to mean IOP in the preoperative, 1-year after surgery, or final follow-up. However, even though none of the eyes in either group had an IOP ≥21 mmHg on the last examination, there was a statistically significant increase in ocular pressure when compared to the two previous time points. The mean IOP in the study and control groups increased from 8.6 ± 1.6 mmHg and 8.5 ± 2.8 mmHg before surgery, respectively, to 15.0 ± 3.7 mmHg and 14.0 ± 3.1 mmHg at the final follow-up. This might be attributed to the physiological change in IOP seen in children as they age^(16)^. A previous study reported a linear increase from 8.0 ± 2.3 mmHg in the normal pediatric population of 0 to 1 years old to 14.9 ± 2.7 mmHg between 11 and 12 years of age^(16)^. Also, the increase in CCT, which is commonly seen after pediatric cataract surgery, might explain some of the changes in measured IOP^(16,17)^. Furthermore, we cannot discard the influence of sedation in underestimating the preoperative and 1-year postoperative measurements to some extent^(18)^.

Due to its potential to permanently impair vision, glaucoma is one of the most feared adverse events following congenital cataract surgery. In children, the disease following cataract procedure is associated with a worse visual prognosis and the need for more medications to control progression as compared to primary congenital glaucoma^(19)^. After a mean of 8 years of surgery, there was no statistically significant difference in glaucoma/glaucoma suspect prevalence between our study and control groups. None of the eyes developed glaucoma, and one eye in each group was considered a glaucoma suspect (9.1% in the study group; 6.7% in the study group).

Recent studies assessing the 5-year follow-up data of congenital cataract surgery reported a prevalence of glaucoma suspect/secondary glaucoma varying from 2.5% to 32.0%^(1,20-23)^. Thus, when compared to oral steroids, intracameral triamcinolone did not statistically increase the prevalence of glaucoma/glaucoma suspect in the long-term follow-up. Moreover, was not associated with a higher prevalence than expected in light of previous reports^(1,20-23)^.

Our study group’s mean age at surgery was 10.9 ± 5.4 months, whereas the control group’s was 10.9 ± 6.1 months, both of which are in the age range associated with decreased secondary glaucoma development^(20,21)^. Although younger age at the surgery has been related to a greater incidence of this complication, especially in children operated on before 7 months old^(20,21-23)^, our glaucoma suspects underwent surgery at 23 and 8 months of age. Furthermore, although many cases of secondary glaucoma occur in the first year following surgery^(1,20)^, the cup-to-disk ratio increase in these two eyes did not occur within this timeframe; thus, they were not identified in our previous paper^(2)^. This supports the evidence that the risk of glaucoma increases over time and reflects the importance of lifelong follow-up for these children^(22,24)^.

In terms of mean CCT, there was a statistically significant increase in both groups on the last examination when compared to the other two timepoints, corroborating previous results of thicker corneas following congenital cataract surgery^(17,25)^. The long-term mean endothelial cell density and central foveal thickness were similarly consistent with other studies in operated children^(26,27)^. The study and control groups had statistically similar mean central pachymetry, endothelial cell density, and central foveal thickness years after surgery, suggesting intracameral preservative-free triamcinolone to modulate postoperative inflammation following congenital cataract surgery has a similar effect on corneal morphology and foveal thickness as oral prednisolone.

The current study’s main limitation is the small sample size achieved in the long-term follow-up. As shown in a previous paper^(28)^, locating patients several years after a procedure is usually challenging. Chougule et al.^(28)^ reported a logarithmic curve of loss to follow-up of children undergoing pediatric cataract surgery, with only 28% attending the 5-year postoperative exam. They discovered that the most important characteristics related to poor compliance with follow-up visits were age at surgery and low economic status. This is consistent with our findings, given that the mean age of our patients at surgery was less than 11 months and they are from the low-income population.

In conclusion, both studied groups had similar ocular findings after a mean of 8 years of congenital cataract surgery, indicating that intracameral triamcinolone acetonide is as safe and effective as oral prednisolone for modulating postoperative inflammation in children younger than two years of age, with the added benefit of facilitating postoperative treatment regimen and compliance.
